# Gold Nanoparticle-Enhanced Molecularly Imprinted Polymer Electrode for Non-Enzymatic Lactate Sensing

**DOI:** 10.3390/bios15060384

**Published:** 2025-06-13

**Authors:** Christopher Animashaun, Abdellatif Ait Lahcen, Gymama Slaughter

**Affiliations:** 1 Center for Bioelectronics, Old Dominion University, Norfolk, VA 23508, USA; 2 Department of Electrical and Computer Engineering, Old Dominion University, Norfolk, VA 23508, USA

**Keywords:** lactate biosensor, laser-induced graphene, gold nanoparticles, molecularly imprinted polymer, PEDOT

## Abstract

We are reporting the development of a high-performance, non-enzymatic electrochemical biosensor for selective lactate detection, integrating laser-induced graphene (LIG), gold nanoparticles (AuNPs), and a molecularly imprinted polymer (MIP) synthesized from poly(3,4-ethylenedioxythiophene) (PEDOT). The LIG electrode offers a highly porous, conductive scaffold, while electrodeposited AuNPs enhance catalytic activity and signal amplification. The PEDOT-based MIP layer, electropolymerized via cyclic voltammetry, imparts molecular specificity by creating lactate-specific binding sites. Cyclic voltammetry confirmed successful molecular imprinting and enhanced interfacial electron transfer. The resulting LIG/AuNPs/MIP biosensor demonstrated a wide linear detection range from 0.1 µM to 2500 µM, with a sensitivity of 22.42 µA/log(µM) and a low limit of detection (0.035 µM). The sensor showed excellent selectivity against common electroactive interferents such as glucose and uric acid, long-term stability, and accurate recovery in artificial saliva (>95.7%), indicating strong potential for practical application. This enzyme-free platform offers a robust and scalable strategy for continuous lactate monitoring, particularly suited for wearable devices in sports performance monitoring and critical care diagnostics.

## 1. Introduction

Lactate is a clinically significant biomarker that reflects tissue oxygenation, metabolic stress, and systemic physiological status. Elevated lactate levels are strongly associated with conditions such as sepsis, ischemia, respiratory failure, and metabolic disorders, and are thus widely used in critical care for early diagnosis, prognosis, and monitoring of treatment response [1,2,3]. Beyond critical care, lactate monitoring has applications in sports medicine, where it is used to optimize athletic training, monitor fatigue, and evaluate endurance thresholds. Despite its clinical and physiological significance, conventional lactate monitoring remains heavily reliant on invasive blood sampling techniques. These methods are often intermittent, labor-intensive, and unsuitable for real-time, continuous assessment, especially in ambulatory or resource-limited settings [4].

To address the limitations of traditional sampling techniques, biosensor technologies have been extensively explored for lactate detection. Enzyme-based electrochemical sensors, particularly those utilizing lactate oxidase (LOx) or lactate dehydrogenase (LDH), represent the most common approach due to their high catalytic efficiency and molecular specificity [5,6,7]. Despite their promise, enzyme-based lactate biosensors suffer from several critical drawbacks including limited thermal and chemical stability, narrow operating pH ranges, and susceptibility to degradation under ambient conditions. These sensors tend to lose significant activity, often exceeding 50% within days of storage at room temperature [8]. Moreover, these enzymes exhibit optimal activity within narrow pH ranges (typically 6.5–7.5), with substantial declines observed outside this window due to conformational changes in their active sites [9]. The poor stability and narrow operating conditions of enzyme-based sensors limit their use, especially for continuous monitoring requiring durability and minimal maintenance [10,11]. Their limited linear range, typically from ten to a few hundred micromolar, fails to cover physiologically relevant lactate levels, which range from 0.5 to 2.5 mM and can exceed 5 mM during sepsis, intense exercise, or ischemia [12,13,14,15].

In response to these challenges, considerable research has focused on developing non-enzymatic lactate sensors that circumvent the limitations of biological recognition elements. Materials such as noble metals (e.g., Pt, Au), transition metal oxides (e.g., NiO, CuO), and carbon-based nanostructures (e.g., graphene, carbon nanotubes) have been employed to enable direct electrochemical detection of lactate via redox reactions or catalytic oxidation mechanisms [16,17,18]. These non-enzymatic sensors offer improved stability, shelf life, and simpler fabrication, but their poor molecular specificity remains a key drawback. Interfering species like ascorbic acid, uric acid, and glucose can cause overlapping signals in complex samples, reducing accuracy. Challenges such as surface fouling, signal drift, and electrode passivation further compromise long-term performance and reproducibility [19,20].

Molecularly imprinted polymers (MIPs) have emerged as an attractive strategy to address the specificity limitations of non-enzymatic sensors. MIPs are synthetic polymer matrices engineered with selective recognition sites that exhibit complementary shape, size, and functional group orientation to a target molecule [21,22]. MIPs offer the dual advantages of synthetic robustness and biomimetic selectivity, making them well-suited for developing stable, reusable, and cost-effective electrochemical sensors. The integration of gold nanoparticles (AuNPs) into MIP-based platforms has gained prominence due to the desirable physicochemical properties of AuNPs, including high electrical conductivity, catalytic activity, and biocompatibility. These characteristics enhance charge transfer kinetics and increase the effective surface area for imprinting, thereby improving sensor sensitivity and responsiveness. Pereira et al. demonstrated a lactate-selective MIP sensor incorporating reduced graphene oxide and AuNPs on a glassy carbon electrode, achieving nanomolar detection limits and excellent selectivity across relevant concentration ranges [23,24]. Building on these insights, the present study employs electrodeposited AuNPs as an interfacial layer on a flexible laser-induced graphene (LIG) electrode to maximize surface activity and provide a conductive scaffold for subsequent molecular imprinting [25,26]. Thereby, paving the way for point-of-care and wearable lactate-based biosensors.

In this work, we report the development of a novel non-enzymatic electrochemical lactate biosensor that integrates LIG, AuNPs, and a poly(3,4-ethylenedioxythiophene) (PEDOT)-based MIP. AuNPs are electrodeposited onto the LIG surface to enhance its electrochemical properties and create a suitable interface for MIP formation. While previous MIP-based lactate sensors have often faced limitations related to dynamic range, reproducibility, or long-term stability, the fabricated sensor design addresses these issues through careful material integration and process optimization. The resulting LIG/AuNPs/MIP sensor demonstrated excellent electrochemical performance: a wide linear detection range from 0.1 µM to 2500 µM, with a low detection limit of 0.035 µM and excellent selectivity towards lactate in the presence of common interfering analytes. Thereby positioning the LIG/AuNPs/MIP sensor as a promising candidate for next-generation flexible biosensors, with practical applications in critical care diagnostics and sports performance monitoring.

## 2. Materials and Methods

### 2.1. Materials

All reagents were of analytical grade and used without further purification. L-lactic acid anhydrous (98%), sodium chloride (NaCl, 99.5%), and potassium phosphate dibasic (K_2_HPO_4_, 99%) were purchased from ThermoScientific (Waltham, MA, USA). Potassium chloride (KCl, 99.0%), sodium phosphate dibasic (Na_2_HPO_4,_ ≥99%), potassium phosphate monobasic (KH_2_PO_4_, ≥99.0%), potassium ferricyanide (K_3_Fe(CN)_6_, 99%), 3,4-ethylenedioxythiophene (EDOT, 97%), gold (III) chloride trihydrate (≥ 49.0% Au basis), acetic acid (CH_3_COOH, ≥99.7%), and calcium chloride (CaCl,99.0%) were purchased from Sigma/Sigma-Aldrich (St. Louis, MI, USA). Sodium bicarbonate (NaHCO_3_, 100.1%) and citric acid anhydrous (C_6_H_8_O_7_, 100.7%) were purchased from Fisher Scientific (Waltham, MA, USA), along with methanol (MeOH). Polyimide (PI–1/2” x 36 Yds 1 Mil Polyimide High-Temperature Heat Resistant Heat Transfer Thermal Electrical Insulation Tape) Tape was purchased from TapeMaster (Troy, MI, USA). Base and Topcoat Nail Polish was purchased from CVS Pharmacy (Norfolk, VA, USA). All solutions were prepared using ultra-pure deionized (DI) water with a resistivity of ≥18.2 MΩ-cm. A total of 63 mM lactate was prepared in 1X PBS (pH 7.4) and EDOT was prepared in 0.1 M LiClO_4_.

Scanning electron microscopy (SEM) measurements were taken using a JSM-IT700HR instrument (JEOL Ltd., Tokyo, Japan). For gold sputtering, MNT-JS1600 sputter was used with DC sputtering at 10 mA for 45 s. Electrochemical measurements were carried out with a PalmSens4 Potentiostat (PalmSens, Houten, Netherlands), linked to a Dell (Round Rock, TX, USA) desktop computer equipped with PS Trace software (v5.9).

### 2.2. Fabrication of Laser-Induced Graphene (LIG) Electrode

**Scheme 1:**
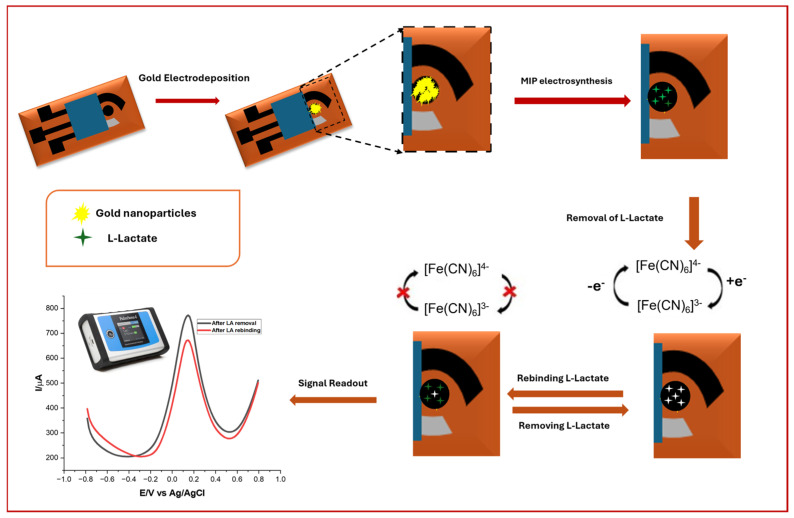
Schematic illustration of sensor development, including deposition of AuNPs and MIP development, along with lactate rebinding.

LIG electrodes were fabricated on commercial polyimide (PI) tape using a BOSS LS1416 CO_2_ pulsed laser engraving system. Laser scribing parameters were optimized through LightBurn software (v0.9.09) to ensure consistent morphology and conductivity of the graphene layer. The laser was operated in fill mode at a speed of 250 mm/s, with a power output of 20% and an engraving interval of 0.1 mm. These conditions were selected to yield a porous and conductive LIG structure suitable for electrochemical applications. Three-electrode configurations—working electrode (WE), counter electrode (CE), and reference electrode (RE)—were patterned directly onto the LIG surface as depicted in Scheme 1. Non-electroactive regions, including electrical leads and interconnects, were passivated with a thin insulating layer of nail polish applied to a defined electroactive area. Following laser processing and passivation, the electrodes were thoroughly rinsed with DI water to remove any residual carbonaceous debris and ensure a clean working surface.

### 2.3. Electrodeposition of Gold Nanoparticles on LIG Electrodes

AuNPs were electrodeposited to enhance the electrocatalytic and surface properties of the LIG working electrode. A 100 µL aliquot of 50 mM HAuCl_4_ aqueous solution was drop-cast onto the WE, and chronoamperometry was performed at −0.7 V for 240 s using a PalmSens4 electrochemical workstation controlled via PSTrace 5.9 software. This deposition process promoted uniform nucleation and growth of AuNPs across the graphene surface. After electrodeposition, the modified electrodes were rinsed with DI water and dried under a nitrogen stream. All procedures were conducted at ambient temperature.

### 2.4. Synthesis of Molecularly Imprinted Polymer (MIP) on LIG/AuNPs Electrodes

Molecular imprinting of lactate onto the LIG/AuNPs-modified electrodes was carried out via electropolymerization of 3,4-ethylenedioxythiophene (EDOT) in the presence of lactate as the template molecule. The concentration of EDOT and the conditions for MIP synthesis, template removal, and square wave voltammetry (SWV) parameters were chosen based on our previously optimized protocol [27]. A 10 mM EDOT solution combined with a 1:10 lactate-to-monomer molar ratio produced the best results regarding film uniformity, electrochemical performance, and selective recognition of lactate. The polymerization solution consisted of lactate and EDOT in a 1:10 molar ratio, optimized to improve template–monomer interaction and subsequent binding site fidelity. A 100 µL volume of the prepolymer solution was drop-cast onto the WE, followed by cyclic voltammetry (CV) performed for 10 cycles in the potential range of −0.7 V to + 1.1 V at a scan rate of 100 mV/s. This process facilitated the uniform deposition of a conductive PEDOT-based MIP film on the AuNP-functionalized surface. Template removal was achieved by immersing the electrodes in a CH_3_COOH:MeOH (3:7, *v*/*v*) solution for 10 min, effectively extracting the embedded lactate molecules and generating specific recognition cavities. The success of template removal was validated through comparative electrochemical analysis.

To evaluate the binding performance of the MIP layer, rebinding studies were conducted by drop-casting 60 µL of lactate solutions with varying concentrations onto the modified working electrode. After a 10 min incubation period at room temperature, SWV measurements were performed to assess the electrochemical response associated with selective lactate recognition. The SWV parameters (1 Hz frequency, 5 mV step potential, 25 mV amplitude) had been previously optimized. All electrochemical experiments were carried out in triplicate to ensure reproducibility and statistical reliability.

For control experiments, non-imprinted polymer (NIP) sensors were fabricated under identical electropolymerization conditions as those of the MIP sensors, but in the absence of lactate in the polymerization solution. PEDOT deposition was conducted via CV over 10 cycles between −0.7 V and + 1.1 V at 100 mV/s. These sensors served as baselines to evaluate non-specific binding and validate the molecular recognition capacity of the imprinted counterparts.

### 2.5. Preparation of Artificial Saliva and Lactate-Spiked Samples

Artificial saliva was prepared following a modified formulation adapted from Morjaria et al. [28], with the detailed composition outlined in Table 1. The solution was prepared to a final volume of 200 mL using DI water. The pH of the mixture was adjusted to 6.7 using 1 M hydrochloric acid to closely mimic the physiological pH of human saliva. To evaluate the sensor’s performance in biologically relevant media, the artificial saliva was subsequently spiked with known concentrations of lactate. Two representative concentrations, 10 µM and 100 µM, were selected to simulate physiological and mildly elevated lactate levels typically encountered in human biofluids under resting and moderate stress conditions. These lactate-spiked saliva samples were used to conduct electrochemical measurements and assess the analytical performance of the LIG/AuNP/MIP-based lactate sensor. All measurements were conducted performed in triplicate to ensure reproducibility.

## 3. Results and Discussion

### 3.1. Optimization of AuNP Electrodeposition for Enhanced Electrocatalytic Performance

**Figure 1:**
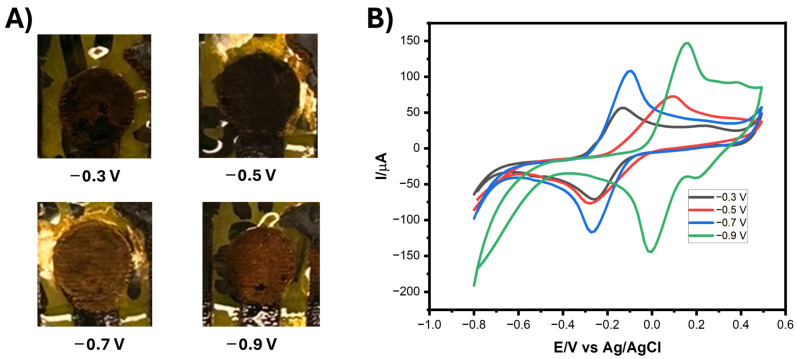
(**A**) Photographs of electrodeposited gold nanoparticles (AuNPs) on laser-induced graphene (LIG) electrodes at varied fixed potentials. (**B**) Cyclic voltammograms (CVs) of the LIG/AuNPs electrodeposited at varied potentials using 5 mM K_3_Fe(CN)_6_ containing 0.1M KCl as the redox probe. Scan rate: 100 mV.s^−1^.

To enhance the electrocatalytic performance of the LIG electrodes, AuNPs were electrodeposited via chronoamperometry (CA) using a well-controlled electrochemical setup. The electrodeposition potential is a critical parameter governing the morphology, size distribution, and surface coverage of AuNPs, all of which influence the sensor’s performance in terms of signal strength, reproducibility, and stability. The corresponding electrodeposition of AuNPs onto the LIG surface at the variant potentials can be visually observed in Figure 1A. As the applied potential increased in magnitude, a corresponding change in the nanoparticle growth profile was observed. The electrochemical behavior of the LIG/AuNPs electrodes was characterized by CV using 5 mM K_3_Fe(CN)_6_ containing 0.1 M KCl as the redox probe. The CV profiles (Figure 1B) revealed that electrodes modified at −0.9 V exhibited the highest peak current. However, this condition also resulted in a pronounced shift in redox potential compared to the bare LIG electrode, indicating a significant alteration in the electrochemical environment. A similar potential shift was observed at −0.5 V. In contrast, deposition at −0.7 V achieved a high current response while preserving the redox potential alignment with the unmodified electrode. These results confirmed that the fixed potential of −0.7 V for 240 s was optimal, offering a balance between sufficient AuNP nucleation and minimal disruption to the electrochemical environment of the underlying LIG. The time duration ensured uniform nanoparticle growth while avoiding overgrowth or aggregation that could impede sensor performance. This condition provided consistent nanoparticle coverage and enhanced redox kinetics, thereby improving surface roughness and accessible catalytic sites. Similarly, others have reported that electrodeposition of AuNPs on LIG surfaces substantially increases peak current and electrocatalytic performance [29,30,31].

### 3.2. Electrochemical Characterization of the LIG/AuNP/MIP Sensor

**Figure 2:**
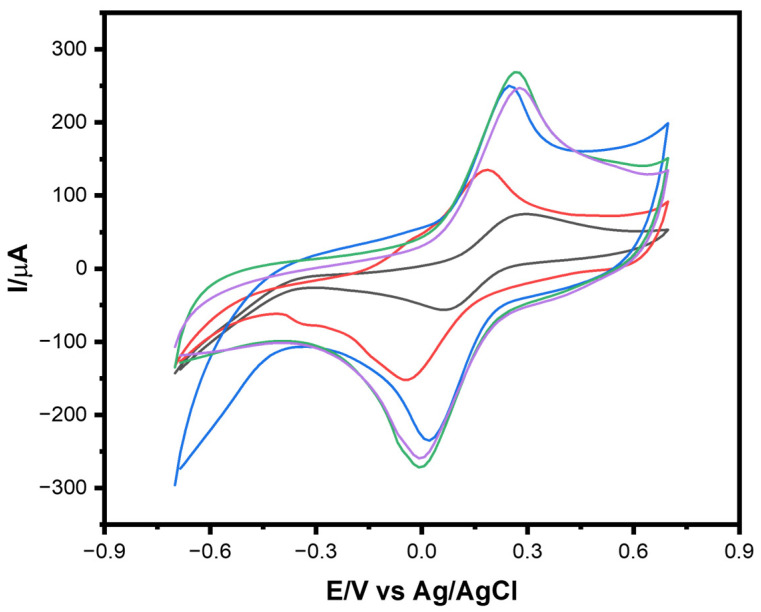
CVs of LIG modified electrodes in 5 mM K_3_Fe(CN)_6_ containing 0.1M KCl: bare LIG (black), LIG/AuNPs (red), LIG/AuNPs with molecularly imprinted polymer (MIP) polymerized (blue), post-template removal (green), and after lactate rebinding (purple). Scan rate: 100 mV.s^−1.^

The sequential fabrication and electrochemical validation of the MIP on the LIG/AuNPs electrode were systematically evaluated using CV, as shown in Figure 2. Each CV profile corresponds to a distinct fabrication step: bare LIG (black), AuNPs-modified LIG (red), LIG/AuNPs/MIP layer post-polymerization (blue), LIG/AuNPs/MIP layer after template removal (green), and LIG/AuNPs/MIP layer following lactate rebinding (purple). This progressive voltammetric characterization provides insight into the electrode’s evolving electrochemical behavior and confirms the success of each functionalization stage. The initial CV of the bare LIG electrode displayed relatively low peak currents, attributable to limited active surface area and suboptimal electron transfer capabilities. Upon electrodeposition of AuNPs (red trace), a marked increase in both anodic and cathodic currents was observed, consistent with the introduction of highly conductive nanostructures that facilitate charge transport and expand the electroactive interface. This result aligns with previous findings demonstrating the catalytic and electron-mediating role of AuNPs in enhancing sensor performance [30,31].

Further enhancement was noted after electropolymerization of a 1:10 ratio solution of lactate to EDOT in the formation of the MIP layer (blue trace). The increased redox peak currents reflect the formation of a porous polymer matrix that retains sufficient electron permeability while beginning to impart selective molecular recognition capabilities. Following template removal (green trace) using a 3:7 ratio solution of CH_3_COOH: MeOH, an additional increase in current was detected, likely due to the exposure of newly formed recognition cavities within the polymer, which improved electrolyte access to the underlying electrode surface. The final step, which involved the re-exposure of the sensor to lactate (purple trace), resulted in a pronounced decrease in redox current. This suppression is attributed to the lactate molecules reoccupying the specific binding sites within the MIP layer, obstructing ion diffusion and electron transfer pathways. The reduction in peak current thus confirms the successful imprinting and recognition of the target analyte, validating the sensor’s target-specific recognition.

### 3.3. Morphological and Elemental Characterization of Electrode Modifications

**Figure 3:**
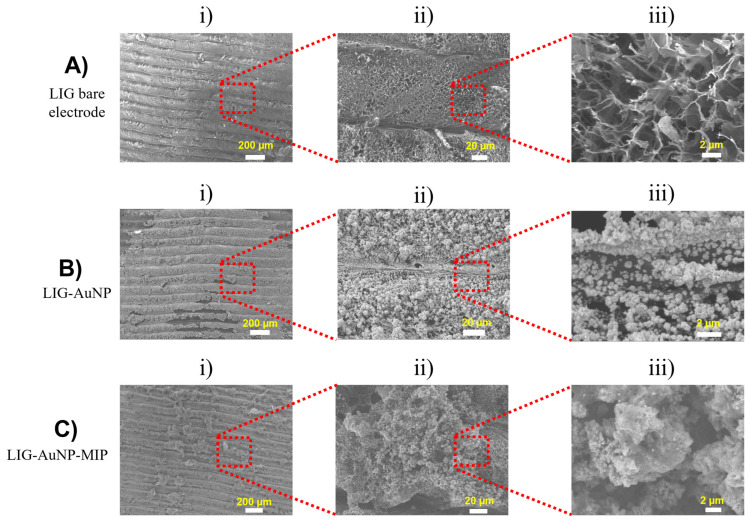
Scanning electron micrographs of electrode surfaces at increasing magnifications (left to right: i) ×70 ii) x550 iii) ×7000). (**A**) Bare LIG, (**B**) LIG/AuNPs, and (**C**) LIG/AuNPs/MIP.

The morphological evolution of the electrode surface across each fabrication stage was investigated using SEM, as illustrated in Figure 3. The micrographs offer a multiscale view, ranging from low to high magnifications, of the surface for (i) the bare LIG electrode, (ii) the AuNPs-modified LIG (LIG/AuNPs), and (iii) the final MIP-functionalized electrode (LIG/AuNPs/MIP). The SEM micrograph of the bare LIG (top row) reveals a characteristic porous carbon network with microscale wrinkling and a hierarchical structure, consistent with literature reports [32,33]. This architecture is highly favorable for electrochemical sensing applications, as it maximizes active surface area and facilitates efficient ion diffusion and electron transport. Upon deposition of AuNPs (middle row), a clear morphological transformation is observed. The LIG surface becomes increasingly populated with densely packed, uniformly distributed nanoparticles. These AuNPs preserve the underlying porosity, enhance surface roughness, and increase electroactive sites, supporting their successful electrodeposition and intimate interaction with the LIG scaffold. ImageJ software’s (v1.54p) quantitative analysis revealed an average AuNP diameter of 87.7 ± 10.47 nm, affirming their nanometric scale and reinforcing the morphological characteristics noted via SEM and EDS.

Further modification with the MIP layer (bottom row) results in a rougher and more granular morphology. The MIP appears as a heterogeneous film conformally coating the AuNPs-decorated surface. This polymeric layer, although slightly obscuring the nanostructural details of AuNPs, preserves the essential porous characteristics of the underlying architecture. The distinct morphological features at each stage affirm the progressive surface functionalization, culminating in a highly textured and chemically tailored interface suitable for selective molecular recognition.

**Figure 4:**
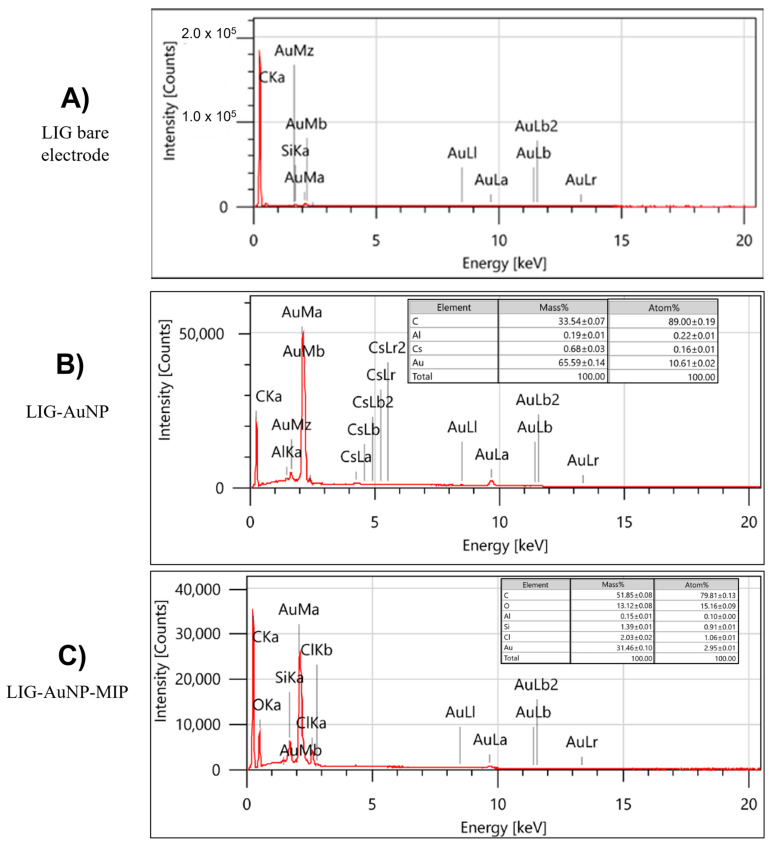
Energy-dispersive X-ray spectra and corresponding elemental composition for the different sensor configurations: (**A**) Bare LIG, (**B**) LIG/AuNPs, and (**C**) LIG/AuNPs/MIP.

Energy-dispersive X-ray spectroscopy (EDS) was employed to assess the elemental composition of the modified electrodes (Figure 4). As expected, the bare LIG surface is overwhelmingly composed of carbon (~99.72%), with trace levels of oxygen and silicon. The latter likely originates from the underlying substrate or sample preparation processes [34,35]. Following AuNPs deposition, EDS spectra reveal the emergence of a strong gold signal, with Au (~65.59%) representing the highest mass percentage among detected elements. This observation corroborates the successful electrodeposition of AuNPs and is consistent with gold’s high atomic weight relative to carbon. Carbon remains the dominant species by atomic percentage, indicating that the bulk material remains LIG. The elemental profile of the final LIG/AuNPs/MIP configuration confirms the continued dominance of carbon and the persistent presence of gold. Importantly, oxygen content notably increases relative to previous stages, likely arising from the polymer matrix used in the MIP formulation. Trace elements such as aluminum, chlorine, and silicon appear in all configurations at low levels, attributable to sample handling, instrumentation, or electrode substrate interactions.

### 3.4. Effect of Scan Rate and Active Surface Area Determination

**Figure 5:**
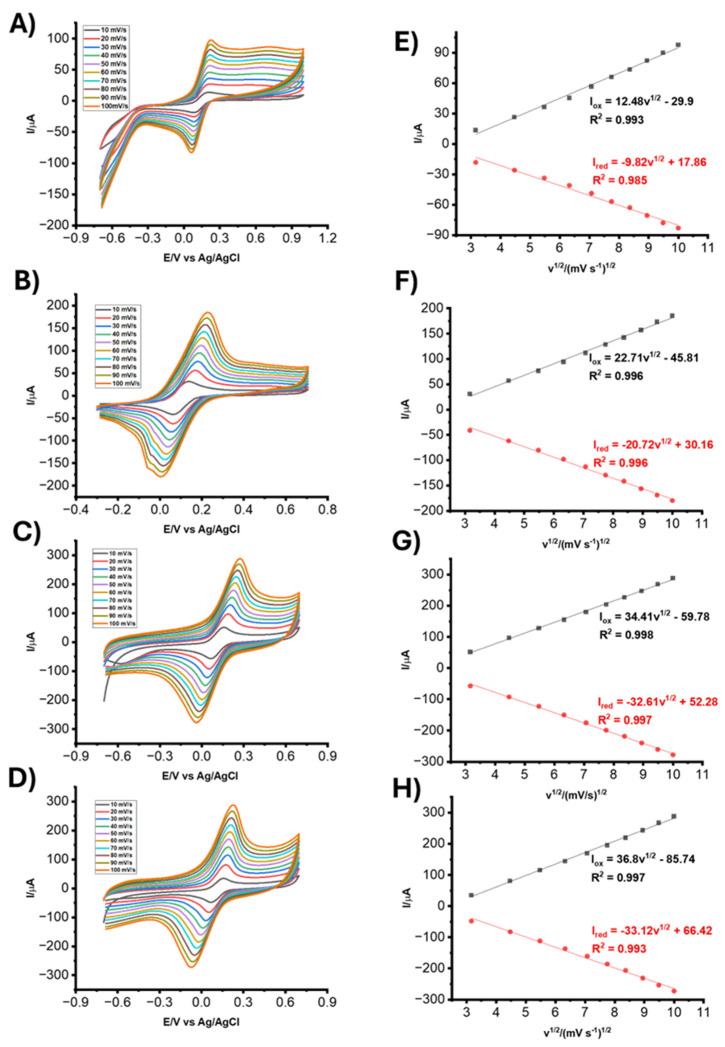
CVs of (**A**) bare LIG, (**B**) LIG/AuNPs, (**C**) LIG/AuNPs/NIP, and (**D**) LIG/AuNPs/MIP electrodes in 5 mM K_3_Fe(CN)_6_ containing 0.1M KCl. Scan rates: 10 to 100 mV.s^−1^. Corresponding I_pa_ vs. *v*^1/2^ for (**E**) bare LIG, (**F**) LIG/AuNPs, (**G**) LIG/AuNPs/NIP, and (**H**) LIG/AuNPs/MIP.

The electrochemical performance and active surface area of the fabricated elec-trodes was evaluated via CV using 5 mM K_3_Fe(CN)_6_ containing 0.1M KCl at scan rates ranging from 10 to 100 mV.s^−1^. Figure 5A–D illustrates the CV responses of the bare LIG, LIG/AuNPs, LIG/AuNPs/NIP (non-imprinted polymer), and LIG/AuNPs/MIP electrodes, respectively. The anodic and cathodic peak currents increase progressively with scan rate for all electrode types, reflecting a diffusion-controlled redox process. Corresponding plots of peak current (I_pa_) versus the square root of scan rate (*v*^1/2^) in Figure 5E–H confirm linearity, further supporting diffusion-controlled electrochemical behavior. Quantitative analysis of the electrochemically active surface area (ESCA) was conducted using the Randles–Sevick equation for a reversible redox couple:

I_pa_ = 268600 n^3/2^ A D^1/2^ C *v*^1/2^ 1) (1)

where *D* is the diffusion coefficient (*D* = 6.70 × 10^−6^ cm^2^ s^−1^), *A* is the active surface area of the electrode, *C* is the concentration of ferricyanide, and *v* is scanning rate (mV.s^−1^), *n* is the number of electrons appearing in the half-reaction for the redox couple and *I*_pa_ is the anodic peak current. Based on the slope of the linear fits of I_pa_ vs. *v*^1/2^ for each electrode, the ECSA was calculated to be 0.0236 cm^2^ for the bare LIG electrode, 0.0351 cm^2^ for the LIG/AuNPs electrode, 0.0444 cm^2^ for the LIG/AuNPs/NIP electrode, and 0.0673 cm^2^ for the LIG/AuNPs/MIP electrode. The progressive increase in electroactive surface area across fabrication stages demonstrates the contribution of both AuNPs and the MIP layer to enhancing the electrode interface. Incorporating gold nanoparticles improves the electron transfer kinetics and introduces nanostructural roughness, while the MIP contributes additional surface complexity and porosity, which collectively result in enhanced electrochemical activity.

### 3.5. Analytical Performance of the LIG/AuNPs/MIP Sensor for Lactate Detection

**Figure 6:**
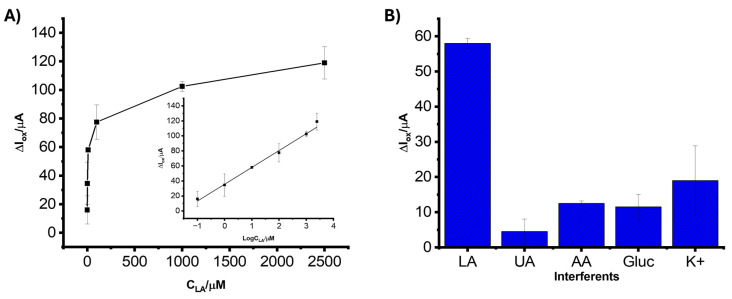
(**A**) Calibration curve showing the relationship between the oxidation peak current (ΔIox) and lactate concentration (C_LA_) ranging from 0.1 µM to 2500 µM. Inset: linear representation of ΔIox versus log(C_LA_). (**B**) ΔI_ox_ of the LIG/AuNPs/MIP biosensor in the presence of 10 µM LA, 100 µM uric acid (UA), 100 µM ascorbic acid (AA), 100 µM glucose (Gluc), and 100 µM potassium chloride (KCl). All measurements were performed in 5 mM K_3_Fe(CN)_6_ containing 0.1 M KCl and in triplicates.

The analytical performance of the LIG/AuNPs/MIP sensor was evaluated via square wave voltammetry under optimized conditions to assess its suitability for lactate detection. As depicted in Figure 6A, the sensor exhibited a clear and proportional increase in oxidation peak current (ΔI_ox_) with increasing lactate concentrations across a wide dynamic range of 0.1 µM to 2500 µM. A strong linear relationship was observed between ΔI_ox_ and the logarithm of lactate concentration (log C_LA_), described by the regression equation:

ΔI_ox_ (µA) = 22.42logC_LA_ + 35.63

This linear regression analysis was characterized by a correlation coefficient (R^2^ = 0.997), which reflects a strong correlation between ΔI_ox_ and log C_LA_ and the sensor’s capacity to quantify lactate accurately over a broad concentration range. The limit of detection (LOD), calculated using the standard 3σ/S method (where σ is the standard deviation of the blank and S is the slope of the calibration curve), was found to be 0.035 µM. This low LOD demonstrates the biosensor’s high sensitivity, attributable to the synergistic enhancement from the AuNPs and the MIP, which together provide a high density of recognition sites and facilitate efficient electron transfer. While most data points displayed low variability, the standard deviation increased slightly at low (0.1 µM) and high (2500 µM) lactate concentrations. This is attributed to signal instability near the detection limit and partial saturation effects at high concentrations, respectively, phenomena commonly observed in electrochemical biosensing platforms.

### 3.6. Selectivity of the LIG/AuNP/MIP Sensor

The selectivity of the LIG/AuNP/MIP sensor toward lactate was assessed in the presence of common electroactive and structurally related analytes. Figure 6B illustrates the current responses obtained from solutions containing 10 µM lactate and interfering compounds, uric acid (UA), ascorbic acid (AA), glucose (Gluc), and potassium chloride (KCl). All measurements were carried out in 5 mM K_3_Fe(CN)_6_ containing 0.1 M KCl. The biosensor exhibited a significantly higher current response to lactate than the other tested analytes. In contrast, UA, AA, Gluc, and KCl generated markedly lower signals, suggesting that the biosensor’s response is not significantly affected by the presence of these analytes. This pronounced discrimination can be attributed to the MIP, which provides selective recognition cavities specifically complementary to the size, shape, and functional groups of lactate molecules. A slight variation was observed in the presence of interferents at 100 µM, as shown in Figure 6B. This variation is attributed to non-specific interactions or ionic disturbances introduced by structurally or electrochemically active species such as uric acid and ascorbic acid. Despite these fluctuations, the current response to lactate remained consistently higher and more stable, reinforcing the high selectivity of the MIP layer.

### 3.7. Stability of the LIG/AuNP/MIP Biosensor

**Figure 7:**
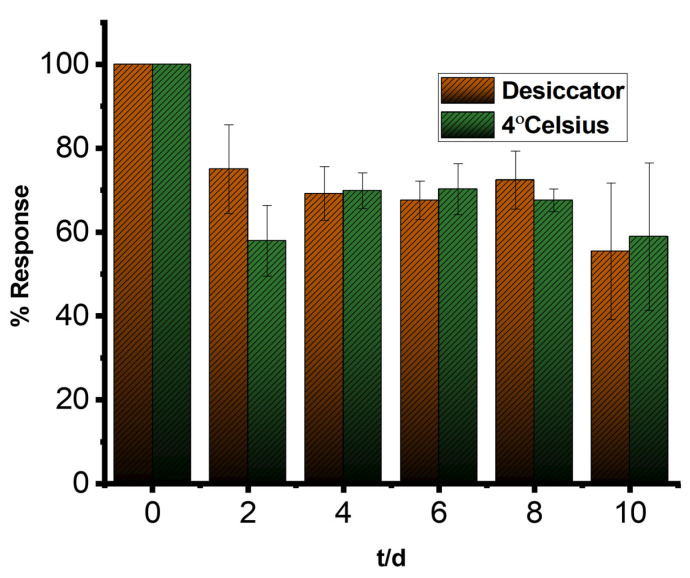
Stability profile of LIG/AuNPs/MIP sensors stored in a desiccator and at 4 °C; measurements were performed using square wave voltammetry at 1 Hz in a 5 mM K_3_Fe(CN)_6_ containing 0.1 M KCl. Measurements performed in triplicate.

Figure 7 presents the results of a stability study comparing the electrochemical performance of LIG/AuNP/MIP sensors stored under two conditions: refrigeration at 4 °C and desiccation at room temperature. Throughout the study period, distinct trends were observed in signal retention and reproducibility. Notably, the biosensors stored in the desiccator exhibited more consistent current responses, as indicated by the narrower error bars, while those stored at 4 °C demonstrated greater signal fluctuations. The observed stability advantage under desiccator storage can be attributed to the reduced exposure to humidity and oxidative conditions, which likely minimized the degradation. This preservation of structural and functional integrity appears to be critical for maintaining the recognition properties of the imprinted sites and sustaining sensor performance. The increase in standard deviation over time, particularly in sensors stored at 4 °C, reflects gradual degradation of the MIP matrix or nanostructured surface. This may result from increased humidity exposure or oxidative effects, which impact the consistency of the sensor’s electrochemical response.

### 3.8. Analytical Performance in Artificial Saliva

The applicability of the LIG/AuNP/MIP sensor in biologically relevant matrices was conducted using artificial saliva spiked with known concentrations of lactate. Artificial saliva was selected as a surrogate for physiological fluids due to its compositional similarity and complexity. Table 2 illustrates the analytical performance of the LIG/AuNP/MIP biosensor in artificial saliva spiked with different lactate concentrations (1 µM to 1000 µM). The biosensor displayed strong recovery rates throughout the tested range, indicating its high accuracy and reliability in complex biofluid matrices. At 1 µM lactate, the sensor achieved a recovery rate of 90.0% and a relative standard deviation (RSD) of 4.44%, demonstrating good precision at the lower detection limit. For intermediate concentrations of 10 µM and 100 µM, recovery values increased to 95.7% and 98.1%, respectively, accompanied by acceptable RSDs of 3.87% and 7.89%. Even at the highest tested concentration of 1000 µM, the sensor upheld a high recovery rate of 93.6% with excellent reproducibility (RSD = 1.47%). These findings validate the sensor’s capability to maintain sensitivity and selectivity across a broad physiological range, even amid potential matrix interferences in artificial saliva. The results confirm the biosensor’s appropriateness for non-invasive lactate monitoring.

A comparative analysis was conducted against previously reported electrochemical sensors for lactate detection, as summarized in Table 3. The performance of the LIG/AuNP/MIP sensor surpasses most enzyme-based and non-enzymatic sensors, including the reported LOx/PB/LIG (LOD: 0.28 µM) [35], PU/LOx/PANI/m-PD/SPAuE (LOD: 7.9 µM) [36], and NiCo LDH (LOD: 0.53 mM) [37]. While a few reported sensors employing noble metal nanostructures, such as AuNPs-ERGO-PAH [38] and MoS_2_-AuPt [39], achieved comparably low LODs, at 1 µM and 0.33 µM, respectively, they operate over narrower concentration ranges and often require more complex fabrication protocols. The ultra-low LOD of the LIG/AuNP/MIP sensor likely results from the synergistic integration of LIG, AuNPs, and MIP. The sensor demonstrated superior performance characteristics, including a wide linear detection range, excellent sensitivity, and strong selectivity. These findings establish the LIG/AuNPs/MIP sensor as a promising platform for sensitive and specific lactate detection in clinical diagnostics and environmental monitoring applications.

## 4. Conclusions

In this study, we developed a highly selective and sensitive lactate biosensor based on LIG electrodes modified with electrodeposited AuNPs and a molecularly imprinted PEDOT layer. The functionally optimized LIG/AuNP/MIP lactate sensor offered significant analytical and practical advancements over existing platforms. The synergistic integration of electrodeposited AuNPs and molecularly imprinted PEDOT enabled a dual-function interface that effectively balances sensitivity, selectivity, and operational simplicity. AuNPs provided enhanced electrocatalytic activity and morphological control, while the MIP layer offered robust target-specific molecular recognition. Collectively, these modifications allowed for lactate detection across an extended broad dynamic range (0.1 µM to 2500 µM) with a high sensitivity of 22.42 µA/log(µM) and LOD of 0.035 µM. The optimized LIG/AuNPs/MIP sensor demonstrated excellent selectivity with minimal interference from common interfering analytes. Future research will assess how buffer composition, pH, and ionic strength influence sensor performance, especially in physiologically varied conditions (e.g., blood plasma) to enhance clinical applicability.

From a translational standpoint, the biosensor’s demonstrated performance in artificial saliva suggests potential for deployment in non-invasive, wearable biosensing devices, validating its suitability for non-invasive, real-time monitoring of lactate in physiological fluids. However, while artificial saliva mimics certain aspects of real biofluids, further validation in clinical matrices (e.g., whole saliva or sweat) and under dynamic physiological conditions is necessary to fully establish robustness and usability.

Analytically, the platform sets a precedent for modular LIG-based biosensing, where surface modifications can be systematically tuned for different targets. The demonstrated strategy of integrating nanoscale particles with imprinting chemistries provides a framework for future non-enzymatic sensors aiming to detect other small metabolites or disease biomarkers. The combination of high sensitivity, broad dynamic range, and excellent selectivity positions the LIG/AuNP/MIP sensor as a promising candidate for clinical, environmental, and physiological lactate monitoring.

## Figures and Tables

**Table 1:** Artificial saliva formulation.

Compound	Concertation	Amount (mg)
KH_2_PO_4_	2.5 mM	68
K_2_HPO_4_	2.4 mM	65.3
NaHCO_3_	15 mM	252
NaCl	10 mM	116.88
CaCl_2_	1.5 mM	33.3
Citric acid	0.15 mM	5.76

**Table 2:** Performance of LIG/AuNP/MIP biosensor in artificial saliva samples.

Sample	Added (µM)	Measured (µA)	Recovery %	RSD %
Artificial Saliva	1	0.9 ± 0.04	90.0	4.44
	10	9.57 ± 0.37	95.7%	3.87%
	100	98.1 ± 7.74	98.1%	7.89%
	1000	936.6 ± 13.8	93.6	1.47

**Table 3:** Comparison of analytical performance of reported biosensors for lactate detection.

Sensor	Technique	Linear Range	LOD	Real Sample	Ref.
LOx/PB/LIG	CA	0.1–30 mM	0.28 µM	N/A	[36]
PU/LOx/PANI/m-PD/SPAuE	CA	0.2–5 mM	7.9 µM	Blood Plasma	[37]
NiCo LDH	CA	5–25 mM	0.53 mM	N/A	[38]
AuNPs-ERGO-PAH	CA	4–16 mM	1 µM	Dairy, Wine	[39]
MoS_2_-AuPt	SWV	5 µM–3 mM	0.33 µM	Sweat	[40]
GCE-Nafion/NiO	CA	0.01–7.75 mM	27 µM	NaOH	[41]
SPCE-NiCo	CA	2–26 mM	5 µM	Sweat	[42]
NH_2_-GP-Cu_3_(BTC) _2_	CV, CA	0.05–22.6 mM	5 µM	Sweat	[43]
Plasma/GNWs/LOx/PET	CA	1 µM–10 mM	1 µM	PBS	[44]
Au/PDA/PtMN	DPV	0.375–12 mM	50 µM	Serum	[45]
PPy/MWCNT	CA	1–15 mM	51 µM	Sweat	[46]
AgNPs/NiO/PU	CA	0.6–2.2 mM	0.27 mM	Plasma	[47]
LIG/AuNP/MIP (This work)	SWV	0.1 µM–2500 µM	0.035 µM	Artificial Saliva	—

Abbreviations: GCE: glassy carbon electrode; NiO: nickel oxide SPCE; NiCo: screen printed carbon electrode, nickel cobalt, ERGO-PAH: electrochemically reduced graphene oxide–poly(allylaminehydrochloride); NH_2_-GP-Cu_3_(btc)_2_: amino-functionalized graphene paper with copper 1,3,5-benzenetricarboxylate (BTC) for metal organic framework (MOF) HKUST-1; LOx: lactate oxidase PB: Prussian blue; PU: polyurethane; PANI: polyaniline; *m*-PD: m-phenylenediamine; SPAuE: screen printed gold electrode; LDH: lactate dehydrogenase; GNWs: graphene nanowalls; PET: polyethylene terephthalate; PDA: polydopamine; PtMN: platinum microneedle; PPy: polypyrrole; MWCNT: multi-walled carbon nanotubes; AgNPs: silver nanoparticles; NiO: nickel oxide; MoS_2_: molybdenum disulfide.

## Data Availability

Data is contained within the article.
